# Study on Low-Frequency Repetitive Transcranial Magnetic Stimulation Improves Speech Function and Mechanism in Patients With Non-fluent Aphasia After Stroke

**DOI:** 10.3389/fnagi.2022.883542

**Published:** 2022-05-30

**Authors:** Guangtao Bai, Liang Jiang, Sai Huan, Pingping Meng, Yuyang Wang, Xiaona Pan, Shuai Yin, Yuyang Zhao, Qiang Wang

**Affiliations:** ^1^Department of Rehabilitation Medicine, The Affiliated Hospital of Qingdao University, Qingdao, China; ^2^Department of Otolaryngology, Qingdao Women’s and Children’s Hospital, Qingdao, China; ^3^Department of Rehabilitation Medicine, Qingdao Women’s and Children’s Hospital, Qingdao, China

**Keywords:** repetitive transcranial magnetic stimulation, aphasia, functional magnetic resonance, BDNF, TNF-α

## Abstract

**Objective:**

To explore the therapeutic effect and mechanism of low-frequency repetitive transcranial magnetic stimulation on the speech function of patients with non-fluent aphasia after stroke.

**Methods:**

According to the inclusion and exclusion criteria, 60 patients with post-stroke non-fluent aphasia were included and randomly divided into treatment group (rTMS group) and sham stimulation group (S-rTMS group). Patients in rTMS group were given low-frequency rTMS + ST training. Patients in the S-rTMS group were given sham low-frequency rTMS + ST training. Once a day, 5 days a week, for a total of 4 weeks. The Western Aphasia Battery and the short-form Token test were used to evaluate the language function of the patients in the two groups before and after treatment. Part of the enrolled patients were subjected to functional magnetic resonance imaging examination, and the morning fasting venous blood of the enrolled patients was drawn before and after treatment to determine the content of BDNF and TNF-α.

**Results:**

In the comparison before and after treatment within the group, all dimensions of the WAB scale of the patients in the rTMS group increased significantly. Only two dimensions of the WAB scale of the patients in the S-rTMS group improved significantly after treatment. The results of the short-form Token test showed that patients in the rTMS group improved significantly before and after treatment. The resting state functional magnetic resonance imaging of the two groups of patients before and after treatment showed: the activation of multiple brain regions in the left hemisphere of the rTMS group increased compared with the control group. The serum BDNF content of the patients in the rTMS group was significantly higher than that of the patients in the S-rTMS group after treatment.

**Conclusion:**

Low-frequency rTMS combined with conventional speech training can significantly improve the speech function of patients with non-fluent aphasia after stroke.

## Highlights

- Low-frequency rTMS can improve the expression and other language functions of patients with non-fluent aphasia after stroke.
- Low-frequency rTMS can promote brain plasticity changes in patients with non-fluent aphasia after stroke.
- Low-frequency rTMS can promote the secretion of BDNF by the central nervous system of stroke patients.

## Introduction

Aphasia refers to a type of language disorder syndrome in which organic brain diseases are caused by various reasons, which cause damage to related brain areas that dominate brain language expression and listening comprehension, so that patients cannot perform normal speech expression and understand the other party’s words. It is very common in patients with cerebrovascular disease. According to research statistics, the incidence of aphasia in stroke patients is about 20–40% (Menichelli et al., 2019).

### Aphasia Recovery Mechanism

Regarding the mechanism of aphasia recovery, when the language hub of the dominant hemisphere is damaged in the acute phase, its inhibition of the surrounding brain areas will be weakened, which promotes the activation of the brain areas around the damaged brain area and the functional reconstruction of plasticity, and promotes the recovery of the patient’s language function. In the subacute phase, the mirror brain area of the language hub of the right hemisphere is activated due to the weakening of the inhibition of the dominant hemisphere, which is beneficial to the recovery of the function of patients with aphasia to a certain extent. In the chronic recovery period, as the function of the dominant hemisphere on the left side of the brain gradually recovers, its activation level gradually increases during language training, and the inhibition to the right hemisphere gradually increases. At the same time, the activation level of the right hemisphere gradually decreased the language hub gradually returns to the left dominant hemisphere. Therefore, in the chronic phase, in order to reduce the inhibitory effect of the non-dominant hemisphere on the dominant hemisphere, it is necessary to inhibit the corresponding brain areas of the non-dominant hemisphere, and at the same time, it can excite the language hub in the dominant hemisphere and promote the recovery of the language function of the patients. Also in clinical practice had showed that cortical stimulation could facilitate functional improvement (Zhang J. et al., 2021).

### Application of rTMS in Aphasia

Repetitive transcranial magnetic stimulation technology is one of the main representatives of non-invasive brain stimulation technology that has emerged in recent years. It not only has a temporary inhibitory or excitatory effect on the cerebral cortex, but also has a long-term plasticity change effect. A large number of research results affirm its efficacy in the treatment of aphasia (Rossetti et al., 2019), but the specific mechanism of action is still unclear. Some scholars use the method of functional magnetic resonance to explore the specific mechanism of the rTMS by the specific activated/inhibited brain regions, but the conclusions are very different (Szaflarski et al., 2018; Arheix-Parras et al., 2021; Fahmy and Elshebawy, 2021; Neri et al., 2021). In addition, studies have also found that: after rTMS treatment, the levels of brain-derived neurotrophic factor in peripheral blood of patients with depression was higher than before, which may be one of the mechanisms of rTMS (Zhao et al., 2019).

### Research Purposes

In this study, low-frequency repetitive transcranial magnetic stimulation was applied to the posterior inferior frontal gyrus of the right cerebral hemisphere in patients with non-fluent aphasia after stroke. Clarify its therapeutic effect on the language function of patients with aphasia, and some patients were enrolled in the rest state functional magnetic resonance scan before and after treatment, using low-frequency amplitude score, degree centrality method to statistically analyze the scanned image data, to identify specific activated or inhibited brain regions, and combined the method of functional connection to explore the plasticity changes of specific brain regions. At the same time, before the start of treatment and after the end of the treatment course, the early morning venous blood of the enrolled patients was collected to determine the content of BDNF, and to explore the treatment mechanism of rTMS in patients with non-fluid aphasia after stroke from the perspective of cytokines, providing clinical and theoretical support for the clinical treatment of aphasia.

## Patients and Methods

### Research Object

According to the inclusion and exclusion criteria, 60 patients with post-stroke aphasia who were hospitalized in the Rehabilitation Medicine Department of Qingdao University Affiliated Hospital from 2017-12 to 2019-10 were randomly divided into treatment group (rTMS group) and control group (S-rTMS group). This study was reviewed by the ethics committee of the Affiliated Hospital of Qingdao University (qyfykyll 2018-23). Written informed consent was obtained from the individual for the publication of any potentially identifiable images or data included in this article.

#### Inclusion Criteria

(1) Clinical compliance with the criteria of “Diagnosis Essentials for Various Cerebrovascular Diseases” formulated by the Fourth National Cerebrovascular Disease Conference of the Chinese Medical Association in 1995; CT or MRI confirmed as the first stroke in the left hemisphere (dominant hemisphere); (2) Right Handy (standardized measurement), normal language function before onset; (3) The course of illness is about 2 weeks to 6 months after stroke; (4) Western Aphasia Battery (WAB) aphasia quotient (AQ) < 93.8, non-Fluent aphasia, with a score of 0–4 for speech fluency; (5) Chinese is the first language, and the education level above elementary school can cooperate to complete the assessment; (6) No epilepsy, severe heart disease, severe physical disease; (7) Clear mind, cooperative physical examination, and orientation Complete, without obvious memory impairment and intellectual impairment; (8) Able to independently maintain a sitting position for more than 30 min; (9) Patients and family members sign informed consent.

#### Exclusion Criteria

(1) Complicated with other neurodegenerative diseases, such as speech disorder caused by Parkinson’s disease, dementia, etc.; (2) Auditory or visual defects may affect assessment and treatment; (3) Application of drugs that change the excitability of the cerebral cortex (antiepileptic drugs), sleeping pills, benzodiazepines, etc.; (4) Combined with epilepsy, severe heart, liver, kidney dysfunction or other serious physical diseases; (5) Unconscious and unable to cooperate with examination and treatment; (6) A history of mental abnormalities; (7) According to safety guidelines, there are contraindications to rTMS and MRI, such as metal foreign bodies in the body or other electronic devices implanted in the body.

Among them, 60 patients completed the initial evaluation of the WAB scale and the short-form Token test. The patients in the rTMS group completed the entire experimental process and completed the final evaluation of the WAB scale and the short-form Token test. One patient in the S-rTMS group was converted due to recurrence of cerebral hemorrhage. He was admitted to neurosurgery for treatment, and another patient was discharged from the trial early due to personal reasons. Therefore, only 28 patients completed the final evaluation of the WAB scale and the short-form Token test. In order to explore the specific mechanism of rTMS, we performed resting functional magnetic resonance scans on some patients before and after treatment, and collected the peripheral blood of the patients, and measured the changes of BDNF and TNF-α in their peripheral serum. The general information of the enrolled patients is shown in the following table (Table 1).

**TABLE 1:** General information of the enrolled patients.

	rTMS group	S-rTMS group
Number of cases	30	30
Clinical scale measurement (pre/post)	30/30	30/28
Serum factor determination (pre/post)	30/28	30/24
fMRI (pre/post)	16/13	12/10
Gender: (Male: Female)	17:13	14:16
Course of disease (months, $\bar{\chi }$ ± s)	3.27 ± 1.50	3.75 ± 1.67
Age (years, $\bar{\chi }$ ± s)	63.47 ± 7.81	59.91 ± 8.58
Aphasia quotient-pre	28.16 ± 22.86	22.76 ± 18.81
Stroke type (hemorrhagic: infarct: mixed) group	13: 15: 2	16: 13: 1

### Research Methods

#### Apparatus

All experiments were completed in the Department of Rehabilitation Medicine and the Central Laboratory of the Affiliated Hospital of Qingdao University.

##### Reagents

Human brain-derived neurotrophic factor (BDNF) enzyme-linked immunoassay (ELISA) kit: enzyme-linked, CK-E12065 human tumor necrosis factor alpha (TNF-α) enzyme-linked immunoassay (ELISA) kit: mlbio, ml077385.

##### Consumables

1.5 ml centrifuge tube (American Axygen), each volume tip (American Axygen).

##### Equipment

Magnetic field stimulator: Wuhan Yiruide Company CCY-IA type.

Electric heating constant temperature blast drying oven: Shanghai Senxin DGG-9140B.

High-speed refrigerated centrifuge: Thermo Scientific.

Pipette: Eppendorf.

Microplate reader: SPECTCA MAX190 (Molecular Company, United States).

#### rTMS Treatment Method

##### Measurement of Motor Threshold

Select the contralateral abductor pollicis brevis muscle as the measuring muscle, and place the recording electrode on the muscle abdomen of the muscle and the reference electrode on the first joint of the thumb of the ipsilateral upper limb. The stimulation coil stimulates the patient’s right brain, gradually adjust the position of the stimulation coil, determine the most suitable stimulation position and stimulation intensity (at this time the incubation period is shortest, and the amplitude is the largest), and gradually adjust the output intensity to find out 10 consecutive stimulations that trigger the contralateral thumb short The stimulus intensity that the abductor motor evoked potential appears at least 5 times and the amplitude is not less than 50 μV is the motor threshold.

##### Stimulation Site

select the patient’s non-dominant hemisphere (right hemisphere) at the back of the inferior frontal gyrus as the stimulation site, place the stimulation coil close to the surface of the patient’s skull and place it tangentially, the center point of the “8” coil is placed at the mark, and the stimulation coil The handle points vertically to the patient’s back occiput. The body surface positioning method is selected according to the electrode positioning map calibrated by the International Electroencephalography Society. Before and after treatment, WAB scale assessment and resting functional magnetic resonance scan were performed on the two groups of patients. The stimulus parameters and stimulus parts of the sham stimulation group were the same as the treatment group, but the stimulation coil was perpendicular to the surface of the skull.

##### Stimulation Parameters

Set 80% of the motor threshold as the stimulus intensity, the stimulus frequency is 1 Hz, 10 pulses are a sequence, the sequence interval is 2 s, 100 sequences per day (total 1,000 pulses in total), treatment for 5 days a week. The total course of treatment is 4 weeks.

#### Routine Speech Training and Language Function Scale Assessment

Conventional speech training is conducted by speech therapists including Schuell training method, blocking removal method, de-inhibition method, program training method and other methods to conduct one-to-one speech training for patients, and appropriately combine computer picture naming training, etc. Each training time is about 30 min. The Western aphasia battery (WAB) Chinese version scale and the short-form Token test were used to evaluate the two groups of patients before and after treatment, and the evaluation results were summarized according to each dimension.

#### Functional Magnetic Resonance Data and Processing Methods

##### Functional Magnetic Resonance Parameter Setting

Use resting functional magnetic resonance (Rs-fMRI) scan for all subjects. The scan parameters are: TR 2,000 ms, TE 30 ms, slice thickness 5.0 mm, no interval, visual The field angle is 240 mm × 240 mm, and the matrix is 960 mm × 960 mm. The imaging range covers the whole brain as much as possible. There are 25 layers from the base of the skull to the parietal lobe, with 279 frames in each layer, and a total of 6,975 images are collected. The acquisition time is 558 s. During the examination, the patient is required to avoid any purposeful thinking activities as much as possible, lie supine on the examination bed with eyes closed, breathe calmly, and keep consciousness. Start scanning after the patient adapts to the magnet and surrounding environment.

##### Image Preprocessing

based on Matlab R2017b platform for preprocessing, and then use DPABI v4.0^1^ and SPM12 software to process the image data. The processing steps are as follows: format conversion, time layer correction, head movement correction, spatial standardization, de-linear shift, regression covariate, etc. Select the 0.010 ∼ 0.027 Hz (slow5) sub-band to process and analyze the image.

##### Fractional Amplitude of Low-Frequency Fluctuation

The fALFF value is the ratio of the sum of the amplitude of the preset frequency band to the sum of the amplitude of the whole frequency band, and then normalize the whole brain voxels, that is, divide by the mean value of whole brain f ALFF to get mfALFF, then Gaussian smoothing (FWHM is 4 mm × 4 mm × 4 mm), you can get the smfALFF result.

##### Degree Centrality Analysis

Each voxel is a node, and the connection between the voxel and the voxel is called an edge. Calculate the Pearson’s correlation coefficient between any two voxels (nodes) with obvious functional connection in the brain function connection group, according to the threshold level of *r* > 0.25, you can get a (number of voxels) × (number of voxels) undirected adjacency correlation matrix, get the weighted DC value, and then divide it with the whole brain DC mean, that is, complete the data standardization process, and then perform Gaussian smoothing for statistical analysis between groups.

##### Functional Connectivity Analysis

Select several speech-related brain regions of interest (ROI) based on previous research at home and abroad, calculate the average time series of each ROI, and then perform pairwise analysis of the above ROI Pearson correlation calculation analysis between, obtain the correlation coefficient between any two ROIs, thus get the correlation matrix, and then normalize it, and then enter the next step of processing and analysis.

#### Serum Processing and Storage Methods

Collect 3 ml of early morning venous blood from the enrolled patients before the treatment and after the end of the treatment course. After centrifugation at room temperature for 5 min, store the centrifuged serum in a refrigerator at −80°C for future reference. If precipitation occurs during storage, it needs to be centrifuged again. Detection steps: balance reagents, prepare reagents, add samples, develop color, terminate the reaction, determine optical density (OD value), use ELISA calc software to calculate serum factor content, etc.

### Statistical Analysis

The scores of the various dimensions of the WAB scale and the short-form Token test score data are analyzed using SPSS19.0 statistical software package. The measurement data obtained in this experiment is expressed as ($\bar{\chi }$ ± s), and the measurement data within and between groups are compared using single Factor analysis of variance, count data using chi-square test, pairwise correlation analysis using linear correlation analysis, *P* < 0.05 indicates that the difference is statistically significant. Use DPABI v4.0 software to perform two-sample *t*-test on the obtained slow5 band fALFF, DC, and FC image data in the rTMS group (after treatment-before treatment) and S-rTMS group (after treatment-before treatment), and use GRF correction to perform multiple Comparative correction, the threshold is individual level *P* < 0.05, clump level *P* < 0.05. Serum BDNF and TNF-α levels were analyzed using SPSS19.0 statistical software package. The measurement data obtained in this experiment are expressed as $\bar{\chi }$ ± s. The measurement data within and between groups are compared by single-factor analysis of variance, *P* < 0.05 indicates a statistical difference.

## Results

(1) The effect of low-frequency rTMS stimulation on the Broca mirror area in the right inferior frontal gyrus on the dimensions of the WAB scale and the short-form Token test scores in patients with non-fluent aphasia after stroke: both the scores of each dimension of the WAB scale in the rTMS group before and after treatment and the short-form Token test scores were significantly improved (*P* < 0.05), while the WAB scale of patients in the S-rTMS group only had three dimensions of spontaneous language, naming, and aphasia quotient, and the short-form Token test scores were significantly improved (*P* < 0.05). After treatment, the scores of the two groups of patients were only statistically different in the three dimensions of WAB scale, spontaneous language, naming, and aphasia quotient (*P* < 0.05), see Table 2 for details.

**TABLE 2:** Evaluation results and statistical analysis of the clinical scale for the two groups of patients.

	rTMS (30)		S-rTMS (28)	
	Pre	Post	Pre	Post
Spontaneous language	5.10 ± 5.03	9.93 ± 5.24*	3.73 ± 4.22	6.68 ± 4.46*^#^
Listening comprehension	3.51 ± 2.50	5.86 ± 2.79*	3.13 ± 2.34	4.69 ± 2.46
Repetition	3.21 ± 2.83	5.56 ± 3.04*	2.69 ± 2.52	3.97 ± 2.91
Naming	2.26 ± 2.41	4.96 ± 2.49*	1.86 ± 2.11	3.39 ± 2.17*^#^
Aphasia quotient	28.16 ± 22.86	52.62 ± 25.02*	22.76 ± 18.81	37.46 ± 20.51*^#^
Short-form Token Test	12.77 ± 8.41	20.90 ± 9.97*	11.17 ± 8.13	17.57 ± 9.19*

*There is a statistical difference in the comparison before and after treatment within the group, P < 0.05.

*^#^Comparison of the two groups after treatment between the groups, there is a statistical difference, P < 0.05.*

**FIGURE 1:**
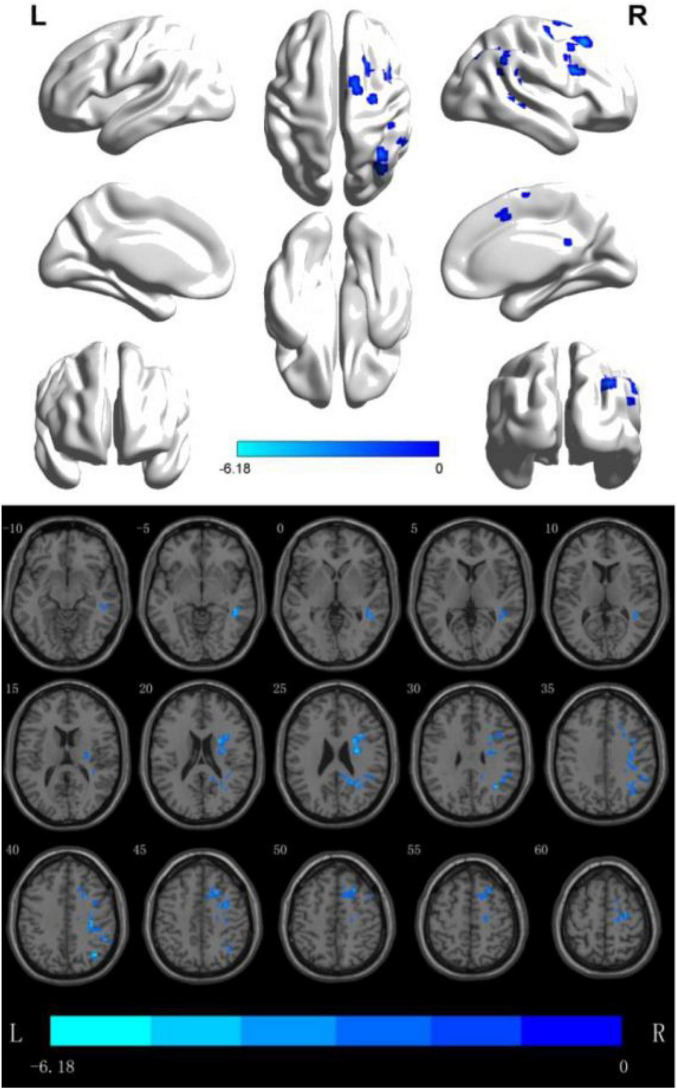
fALFF analysis of the difference between the two groups before and after treatment. The blue area is the brain area where activation is inhibited (threshold: individual level *P* < 0.05, clump level *P* < 0.05).

**FIGURE 2:**
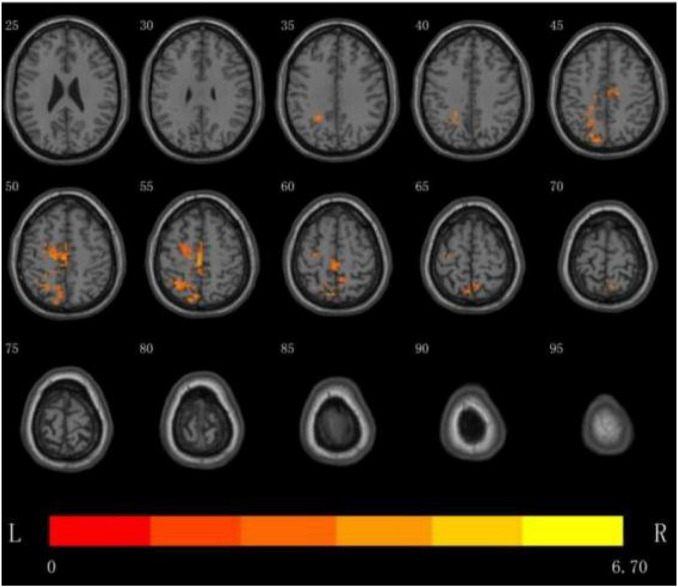
DC analysis of the distribution of brain regions with significant differences between the two groups before and after treatment, the yellow area is the activated brain area (threshold: individual level *P* < 0.05, clump level *P* < 0.05).

**FIGURE 3:**
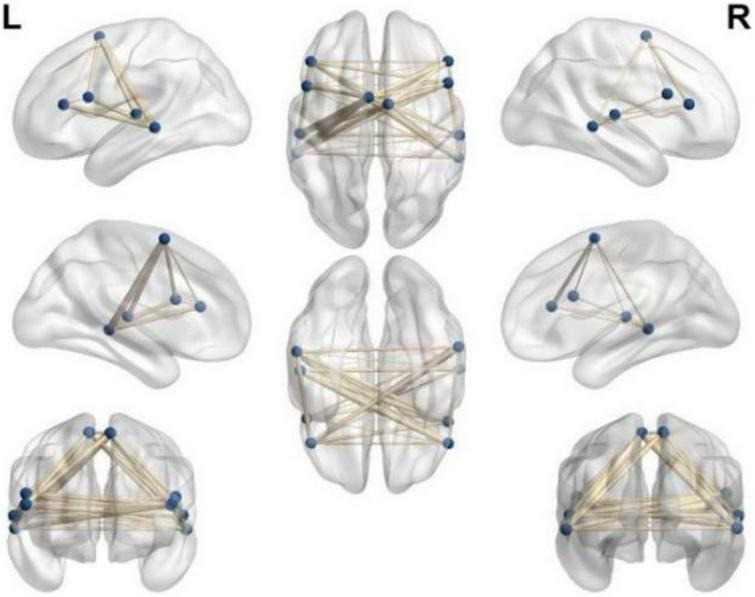
Select ROI function connection pairwise comparison function connection diagram (*p* < 0.05).

(2) fALFF analyzes the brain regions where the difference between the two groups is more meaningful: through data analysis, it can be seen that in the Slow5 subband, there are two Clusters with statistical differences, The fALFF value of multiple brain regions of the patients in the rTMS group was decreased than that of the patients in the S- rTMS group, such as the right dorsolateral superior frontal gyrus, right supplementary motor area, right inferior frontal gyrus pars opercularis (voxel 56, MNI *X* = 36, *Y* = −39, *Z* = 15, *T* = −4.76, *P* < 0.05), right Brodmann area 8, right angular gyrus, right supramarginal gyrus, and right middle temporal gyrus (voxel 19, MNI *X* = 27, *Y* = −9, *Z* = 24, *T* = −5.37, *P* < 0.05) indicating that the activation of the above brain regions in the rTMS group was suppressed than that of the patients in the S-rTMS group. See Figure 1 for details.

(3) DC analysis of the brain regions where the difference between the two groups is more meaningful: the results show that the DC value of multiple brain regions of the patients in the rTMS group was enhanced than that of the patients in the S- rTMS group, such as the left parietal lobe [superior parietal lobule (voxel 78, MNI *X* = −12, *Y* = −81, *Z* = 48, *T* = 4.74, *P* < 0.05), angular gyrus)], left frontal lobe [BA6 area, middle frontal gyrus, superior frontal gyrus, supplementary motor area (voxel 35, MNI *X* = −3, *Y* = −24, *Z* = 57, *T* = 6.70, *P* < 0.05), paracentral lobule], bilateral Limbic lobe (cingulum gyrus) indicating that the activation of the above brain regions in the rTMS group was significantly higher than that of the patients in the S-rTMS group. See Figure 2 for details.

(4) On the basis of the previous image processing, according to the pre-selected multiple ROIs related to the language function, the pairwise function connection analysis is performed on the basis of the difference between the two groups, and the *t*-test is performed. The result shows: between the left frontal lobe (supplementary motor area) (voxel 35, MNI *X* = −3, *Y* = −24, *Z* = 57, *T* = 6.70, *P* < 0.05) and the right temporal lobe (middle temporal gyrus) (voxel 19, MNI *X* = 27, *Y* = −9, Z = 24, *T* = −5.37, *P* < 0.05) became stronger (*P* < 0.05), indicating that the connection between the two hemispheres of the patients in the rTMS group was strengthened as shown in Figure 3.

(5) Changes in the serum BDNF content of the two groups of patients before the treatment and after the end of the treatment course: the serum BDNF content (pg/ml) of the patients in the rTMS group increased from 35.34 to 42.09 (*P* < 0.05), while the serum BDNF of the patients in the S-rTMS group the content (pg/ml) increased from 31.24 to 34.76 (*P* > 0.05). There was no significant difference in serum BDNF content between the two groups before treatment (*P* > 0.05). After the treatment, the serum BDNF content of patients in the rTMS group was significantly higher than that of the patients in the S-rTMS group (*P* < 0.05) and the specific results are shown in Table 3.

**TABLE 3:** Comparison statistical scores of serum BDNF levels before and after treatment in the two groups.

	Number pre/post	Pre-BDNF (pg/ml)	Post-BDNF (pg/ml)	*P-*value in group
rTMS group	30/28	35.34 ± 5.53	42.09 ± 9.16	0.0116
S-rTMS group	30/24	31.14 ± 9.27	34.76 ± 8.79	0.4326
*P*-value between groups		0.2541	0.0077	

## Discussion

Aphasia is an organic brain disease caused by various reasons, causing damage to the related brain areas that dominate brain language expression and listening comprehension, which leads to a language disorder syndrome with abnormal speech expression and abnormal listening comprehension. Among the various diseases of brain damage, stroke is the most common. According to research statistics, about 30% of stroke patients are accompanied by aphasia (Menichelli et al., 2019).

As a new non-invasive technology that directly acts on the cerebral cortex, rTMS has been proven by many studies to treat patients with aphasia after stroke, but its specific mechanism is still controversial.

In this study, patients with non-fluent aphasia after stroke were treated with low-frequency rTMS for 4 consecutive weeks. The WAB scale and short-form Token test were used to evaluate the aphasia of patients before and after the treatment. The results of the study found that the low-frequency rTMS on the posterior inferior frontal gyrus of the right hemisphere combined with conventional speech training compared with false low-frequency rTMS combined with conventional speech training, the patient’s naming, spontaneous language and other expression skills have been significantly improved.

Low-frequency rTMS combined with speech training can significantly improve the expression ability of patients with aphasia. The results of this study are consistent with the conclusions of previous studies. Some scholars use low-frequency rTMS as a single treatment method. After a short-term treatment is given to the patient’s non-dominant hemisphere inferior frontal gyrus (the mirror area of the Broca area) for a short period of time, it is found that this single stimulation can improve the accuracy of patient naming, and the patient’s reaction time will be significantly shortened (Terao and Ugawa, 2002).

Harvey et al. (2019) used another continuous theta pulse magnetic stimulation (similar to low-frequency rTMS stimulation) to act on the posterior part of the right inferior frontal gyrus of patients with chronic aphasia. After treatment, they found that the patient’s picture naming ability was significantly improved, indicating that this treatment plan is beneficial to improve the patient’s naming ability (Harvey et al., 2019). Some scholars also use low-frequency rTMS to stimulate the posterior part of the inferior frontal gyrus of the non-dominant hemisphere for 10 times. The results show that this treatment can significantly improve the patient’s language fluency (Lopez-Romero et al., 2019). Some scholars have also combined low-frequency rTMS on the posterior part of the right inferior frontal gyrus with speech training. After 2 weeks of treatment, the language fluency of patients with aphasia in the treatment group has improved greatly compared with the control group (Haghighi et al., 2017).

Our study combined low-frequency rTMS with conventional speech training and found that its therapeutic effect was significantly better than that of simple speech training. There are many similar studies. Yoon et al. (2015) combined low-frequency rTMS therapy with speech therapy to explore the combination of the two and the therapeutic effect. The treatment course was 4 weeks. The Korean version of the WAB scale was used to evaluate the two groups of patients before and after treatment. It was found that the naming and retelling ability scores of the patients after low-frequency rTMS stimulation increased significantly, and rTMS combined with speech training can be used as a treatment for brain Effective treatment for patients with non-fluent aphasia after stroke (Yoon et al., 2015).

In order to explore the specific mechanism of low-frequency rTMS on patients with aphasia, we performed resting functional magnetic resonance examinations on some patients before and after treatment, and used different analysis methods to perform statistical analysis on the obtained image data. First, use the fractional Amplitude of Low-frequency Fluctuation (fALFF) method to analyze. This method is to standardize the ALFF at the individual level. After the standardization at the individual level, the shortcomings of ALFF can be effectively avoided, and the sensitivity and specificity of detection can be improved. Some scholars (Liu, 2016) used functional magnetic resonance to study the brain plasticity of patients with aphasia and found that rehabilitation training can increase the ALFF value of the temporal lobe of the left cerebral hemisphere and the right cerebellum, suggesting that the above brain areas are in the recovery process of aphasia play an important role.

Our study found that the fALFF value of multiple brain regions in the right hemisphere frontal lobe (right inferior frontal gyrus pars opercularis, right supplementary motor area, etc.) decreased. It shows that the activation of the above brain regions was significantly inhibited in the rTMS group of patients. The reason for the analysis is that low-frequency rTMS treatment on the mirror area of the Broca area of the right regions can inhibit the activation of this area, showing the above-mentioned hypoperfusion in the brain regions, and the local blood oxygen is disproportionately reduced due to the decreased oxygen consumption of neurons. Deoxyhemoglobin (paramagnetic) is relatively increased, so it shows a weakened signal. This shows that low-frequency rTMS can indeed significantly inhibit the ROI of the target area, thereby reducing the activation of the right brain area, reducing its inhibition of the Broca area of the dominant hemisphere through the corpus callosum, promoting the activation of the Broca area of the dominant hemisphere, and improve the speech expression functions of patients with aphasia by promote the brain plasticity. Some scholars have found that the degree of language impairment in patients with aphasia is positively correlated with the Pearson correlation test of the right middle frontal gyrus (Zhu et al., 2014), which is consistent with our study that the activation of the right frontal lobe decreases and the activation of the left inferior frontal gyrus increases during the recovery period of speech function.

At the same time, the activation of the right temporal lobe (middle temporal gyrus) and right parietal lobe (corner gyrus, right superior marginal gyrus) and other brain regions in the rTMS group was also inhibited. The analysis reason was considered to be given to the right inferior frontal gyrus with low frequency after rTMS treatment, the activation of this area decreases, and at the same time, there are different degrees of functional connection between this area and the surrounding brain areas. The function decline of a brain region will also affect the function of the surrounding brain regions. The function of the above-mentioned regions in the right hemisphere decreases, and the function of the above-mentioned regions will weaken the inhibitory effect to the corresponding brain regions of the dominant hemisphere, thereby promoting the functional activation and recovery of the above-mentioned brain regions in the dominant hemisphere. As the above brain regions in the dominant hemisphere are the reading center and the naming center, it can also explain why the low frequency rTMS stimulated on the posterior part of inferior frontal gyrus in the right hemisphere can improve naming and dyslexia in patients with aphasia.

Then, we used the Degree Centrality (DC) method to analyze. DC reflects the number of connections in the adjacent areas of the brain. Specifically, it refers to the number of direct connections between a node in the brain and other adjacent nodes, which can be directly quantified (Van den Heuvel and Sporns, 2013). DC can reflect the attributes of important nodes (hub nodes) at the center of the brain network. Because of its high connectivity with the surrounding brain nodes, it has a core dominance, and even has long-distance connections with other nodes, and its functions are the most complex, so its energy consumption (oxygen consumption) is higher than that of general nodes, also it is easy to be damaged in cerebrovascular diseases (Bullmore and Sporns, 2012). Wise (2003) found that speech training can activate the brain areas around the damaged language center in the dominant hemisphere. Other studies believe that inhibiting the activation level of the right cerebral hemisphere and reducing its inhibition to the dominant hemisphere through the corpus callosum can improve the long-term efficacy of patients with aphasia (Breier et al., 2009).

The left triangular of the inferior frontal gyrus in the dominant hemisphere is known as the classic language brain area of “oral expression,” which is mainly used for speech planning and execution. In recent years, scholars believe that the scope of the classic Broca area should include other areas of the frontal lobe, such as the frontal middle gyrus of the dominant hemisphere which responsible for participating in language production. At the same time, studies have found that the superior hemisphere superior frontal gyrus is also a key area in the language network, which is related to patients’ language fluency and functions such as semantic conversion, retelling, naming, and listening comprehension (Sollmann et al., 2014). The results of our study showed that the DC value of the brain regions was increased such as the left superior parietal lobule, the left angular gyrus, left frontal lobe (BA6 area, middle frontal gyrus, superior frontal gyrus, supplementary motor area, paracentral lobule), bilateral Limbic lobe (cingulum gyrus) indicating that the above brain regions of the patients in the rTMS group were activated compared with that of the patients in the S-rTMS group, which also supports the above view.

Finally, we adopt the method of Functional Connection (FC) to analyze. FC reflects the degree of connection of neuronal activity between different brain regions that are far away. Through rs-fMRI, the functional network and anatomical structure of the entire brain can be studied. Li (2017) studied the brain function of patients with motor aphasia after stroke and found that after 1 month of rehabilitation, the functional connection between the middle temporal gyrus of the left dominant hemisphere and the left frontal lobe, insula and other brain regions increases. At the same time, the functional connection between the middle temporal gyrus of the left dominant hemisphere, the marginal lobe of the left hemisphere, and the cerebellum decreased. During the recovery period of aphasia, the functional connection between the left middle frontal gyrus and the undamaged brain area around the damaged brain area also increases. In addition, some researchers believe that (Zhu et al., 2017), patients with acute stroke not only have disordered language central function, but also interfere with the default network of the brain, which leads to a decline in the cognitive function of stroke patients.

Some scholars (Sreedharan et al., 2019) found that the recovery of language function in patients with aphasia after stroke is often accompanied by changes in functional connectivity: in the acute phase, the functional connection coefficient of the language neural network is significantly reduced. In the chronic phase, the functional connection coefficient of the language neural network is significantly enhanced. The study also found that even in high-risk patients, there is a decrease in resting functional connectivity. Some scholars (Zhang C. et al., 2021) found in the study of patients with motor aphasia after stroke that the patients were accompanied by a significant decline in language ability, and the average functional connectivity index of the frontal and parietal lobe in the left dominant hemisphere also decreased significantly. As language comprehension improves, and the average connection index of the frontal and parietal lobe of the dominant hemisphere also gradually increases. The above phenomenon shows that the language comprehension ability of patients with aphasia after brain injury improves, it may be achieved by changing the functional connections between brain areas. Moreover, research has confirmed that the improvement of language function in patients with aphasia is also related to the changes in the functional connections of brain regions.

The supplementary motor area is very important for motion control. The front area of the supplementary motor area is mainly responsible for the preparation and selection of sports. The back of the supplementary motor area is responsible for the execution of the movement, and the entire supplementary motor area plays a decisive role in both the low-level execution of the movement and the high-level control of the movement. Studies have confirmed that the treatment of bilateral supplementary motor area in patients with aphasia can improve the naming ability of patients (Naeser et al., 2020). This also indicates that the treatment of low-frequency rTMS on the mirror area of the Broca area in the right hemisphere can improve the language function of patients with aphasia by improving the number and efficiency of functional connections in multiple brain areas. Our research also found that after low-frequency rTMS treatment, the functional connection between the supplementary motor area of patients with aphasia and some brain regions of the bilateral hemispheres was significantly enhanced, and this may be one of the possible mechanisms for low-frequency rTMS to improve patients with aphasia.

At the same time, in order to explore the specific therapeutic mechanism of low-frequency rTMS, we measured the changes in peripheral serum BDNF and TNF-α concentrations of the enrolled patients before and after treatment, and planned to explore the specific mechanism from the perspective of cytokines. Studies have confirmed that there are many nutritional factors in the brain to promote the recovery and improvement of brain function in patients. After rTMS treatment, it may promote the release of some nutritional factors in the brain and promote the repair or improvement of damaged brain function (Arheix-Parras et al., 2021).

Studies have found that after rTMS treatment, the levels of BDNF in peripheral blood of patients with depression are higher than before, which may be one of the mechanisms of rTMS (Zhao et al., 2019). So, we detected the serum BDNF concentration in peripheral blood of the two groups of patients before and after treatment.

It is well known that BDNF is essentially a protein, which plays an important role in the growth and differentiation of nerve cells, and can repair damaged nerve cells, thereby improving advanced cognitive functions (learning, memory, etc.) (Asadi et al., 2018; Huey Fremont et al., 2020), especially, it plays an important role in mediating the neural plasticity process of language function recovery in patients with aphasia after stroke (Di Pino et al., 2016). Animal studies have also shown that BDNF promotes long-term potentiation (LTP) through TrkB signaling (Lamb et al., 2015), which is considered to be essential for the intermittent memory process of the hippocampus (Zagrebelsky and Korte, 2014). Moreover, studies have found that BDNF can cross the blood–brain barrier through a high-volume saturated transport system. Animal studies have observed a positive correlation between the levels of BDNF in the brain and blood (Angelucci et al., 2011). Morichi et al. (2013) also found that changes in BDNF in peripheral blood are related to changes in cerebrospinal fluid (CSF) BDNF, and changes in BDNF at the peripheral level may reflect changes in BDNF in the brain. Therefore, some scholars believe that changes in BDNF at the peripheral level may reflect or at least partially reflect changes in brain BDNF (Fritsch et al., 2010). Winter et al. (2007) also found that the serum BDNF content increased significantly after high-intensity exercise, and the vocabulary learning speed was also significantly improved. They believed that the increase in the short-term learning success rate was related to the increase in the BDNF level.

Therefore, in this study, the peripheral blood of patients in the enrolled group was collected before and after low-frequency rTMS treatment, and the changes in peripheral serum BDNF content were measured, and then to explore the BDNF content changes in central nervous system. The results showed that the serum BDNF content of peripheral blood in the rTMS group increased significantly before and after treatment, and the serum BDNF content of the patients in the rTMS group was significantly higher than that of the patients in the S-rTMS group after treatment. This suggests that patients with non-fluent aphasia have a significant increase in serum BDNF levels in peripheral blood after low-frequency rTMS treatment. Many previous studies have confirmed that the increase in serum BDNF content in peripheral blood can reflect the changes in BDNF content in the patient’s brain from the side. Therefore, we believe that after low-frequency rTMS stimulation, the content of BDNF in the brain of patients also increases to a certain extent, which may be one of the mechanisms of low-frequency rTMS.

In summary, our research results show that low-frequency rTMS combined with conventional speech training can significantly improve the language function of patients with non-fluent aphasia. In addition to directly changing the excitability of the cortex of the stimulated brain area, rTMS can inhibit the activation of different brain areas in the frontal and temporal lobes of the right cerebral hemisphere, and promotes the activation of different brain regions in the frontal and temporal lobes of the left dominant hemisphere, thereby improving the function of different brain regions and promoting changes in brain plasticity. It will also affect the transmission, expression and release of various cytokines and neurotransmitters, especially the expression and release of BDNF, which in turn promotes changes in brain plasticity. This may be one of the mechanisms by which rTMS promotes the improvement of the central nervous system, especially the language function of the brain.

## Data Availability Statement

The original contributions presented in the study are included in the article/supplementary material, further inquiries can be directed to the corresponding author.

## Ethics Statement

The studies involving human participants were reviewed and approved by the Ethics Committee of The Affiliated Hospital of Qingdao University. The patients/participants provided their written informed consent to participate in this study.

## Author Contributions

QW and GB: conceptualization. LJ: methodology and formal analysis. YW: software. PM and XP: validation. SY and YZ: investigation. SH: resources and data curation. GB: writing—original draft preparation. GB, LJ, and QW: writing—review and editing. All authors have read and agreed to the published version of the manuscript.

## Conflict of Interest

The authors declare that the research was conducted in the absence of any commercial or financial relationships that could be construed as a potential conflict of interest.

## Publisher’s Note

All claims expressed in this article are solely those of the authors and do not necessarily represent those of their affiliated organizations, or those of the publisher, the editors and the reviewers. Any product that may be evaluated in this article, or claim that may be made by its manufacturer, is not guaranteed or endorsed by the publisher.

## Footnotes

1
http://rfmri.org/dpabi
